# Federal opioid agonist therapy policy: interrupted time series analysis of the impact of the methadone exemption removal across eight provinces in Canada

**DOI:** 10.1186/s12913-024-11281-9

**Published:** 2024-08-05

**Authors:** Abhimanyu Sud, Chloe Campbell, Arani Sivakumar, Ross Upshur, Rahim Moineddin, Kellia Chiu

**Affiliations:** 1 Temerty Faculty of Medicine, University of Toronto, Toronto, Canada; 2 Humber River Health, Toronto, Canada; 3 Schulich School of Medicine & Dentistry, Western University, London, Canada; 4 Lunenfeld-Tannenbaum Research Institute, Sinai Health System, Toronto, Canada; 5 Dalla Lana School of Public Health, University of Toronto, Toronto, Canada; 6 School of Pharmacy, Faculty of Medicine and Health, The University of Sydney, Sydney, Australia

**Keywords:** Opioid-related disorders, Opioid substitution treatment, Opioid epidemic, Methadone, Health policy, Interrupted time series analysis, Canada

## Abstract

**Background:**

Federal deregulation of opioid agonist therapies are an attractive policy option to improve access to opioid use disorder care and achieve widespread beneficial impacts on growing opioid-related harms. There have been few evaluations of such policy interventions and understanding effects can help policy planning across jurisdictions.

**Methods:**

Using health administrative data from eight of ten Canadian provinces, this study evaluated the impacts of Health Canada’s decision in May 2018 to rescind the requirement for Canadian health professionals to obtain an exemption from the *Canadian Drugs and Substance Act* to prescribe methadone for opioid use disorder. Over the study period of June 2017 to May 2019, we used descriptive statistics to capture overall trends in the number of agonist therapy prescribers across provinces and we used interrupted time series analysis to determine the effect of this decision on the trajectories of the agonist therapy prescribing workforces.

**Results:**

There were important baseline differences in the numbers of agonist therapy prescribers. The province with the highest concentration of prescribers had 7.5 more prescribers per 100,000 residents compared to the province with the lowest. All provinces showed encouraging growth in the number of prescribers through the study period, though the fastest growing province grew 4.5 times more than the slowest. Interrupted time series analyses demonstrated a range of effects of the federal policy intervention on the provinces, from clearly positive changes to possibly negative effects.

**Conclusions:**

Federal drug regulation policy change interacted in complex ways with provincial health professional regulation and healthcare delivery, kaleidoscoping the effects of federal policy intervention. For Canada and other health systems such as the US, federal policy must account for significant subnational variation in OUD epidemiology and drug regulation to maximize intended beneficial effects and mitigate the risks of negative effects.

**Supplementary Information:**

The online version contains supplementary material available at 10.1186/s12913-024-11281-9.

## Background

Globally, people living with opioid use disorder (OUD) continue to face challenges with accessing opioid agonist therapy (OAT), despite demonstrated improvements along a range of outcomes from social functioning to mortality [1]. A multitude of structural barriers such as various kinds of stigma, poor coordination of substance use care with primary care, inequitable remuneration for health professionals, and a dearth of health professions training have been identified as contributing to these access challenges [2, 3].

Perhaps chief among these is the over-regulation of agonist therapies, primarily in the forms of methadone and buprenorphine, both at federal (e.g. through drug control legislation) and local levels (e.g. through health professions regulations). Many have called out an apparent irrationality of tight regulation of methadone and buprenorphine in the face of comparatively little regulation of full-agonist opioid analgesics like oxycodone and fentanyl. As such, in several countries, policies to deregulate OAT with aspirations of expanding access have been implemented to varying degrees. In the early 2000s, France removed buprenorphine prescribing regulations, permitting French physicians to prescribe buprenorphine without requiring the completion of special education or licensing [4]. The apparent success of this policy change has inspired advocacy for deregulation internationally [5]. For example, in early 2023 the United States eliminated the need for health professionals to acquire an X waiver to prescribe buprenorphine for OUD management. This amendment permits US health professionals with a standard Drug Enforcement Administration controlled medication license to prescribe buprenorphine without any federal patient caps, though still being subject to state requirements [6]. An explicit aspiration of this policy intervention is to increase the number of OAT prescribers and thus improve access to effective OUD care in the context of ever-growing opioid-related harms [7].

Thus far, there has been little evaluation of such regulation-focused policy maneuvers on either proximal (e.g., numbers of prescribers) or distal (e.g., access to treatment and opioid-related harms) outcomes. Importantly, other strategies to increase the OAT prescribing workforce such as extending prescribing privileges to nurse practitioners and physician assistants have resulted in important access expansions [8], though in the US these expansions have disproportionately privileged white and higher-income people [9–11]. Given the rising global prevalence of OUD [12–14], other countries may also engage in similar policy planning and can benefit from evaluations of comparable policies.

On May 19, 2018, Health Canada rescinded the Sect. 56 exemption requirement from the *Controlled Drugs and Substances Act*, a federal requirement for health professionals to be able to sell, provide, or administer methadone [15]. A national consultation had identified this exemption requirement as a structural barrier to OAT, and an explicit aspiration of this policy intervention was to increase the OAT prescribing workforce and improve effective OUD care access nationally, in a similar context as the US of dramatically increasing opioid-related harms. Buprenorphine (including both buprenorphine-only formulations and co-formulation with naloxone), as the other primary pharmacological treatment for OUD in Canada, was never subject to such direct federal regulation in Canada. This policy change thus provides an opportunity to assess the impact of federal deregulation policy on the prescriber workforce and inform policy learning in comparable jurisdictions. A focus on methadone is particularly pertinent as the increasing potency and toxicity of the drug supply in North America has highlighted the ongoing utility of higher potency, full agonists pharmacotherapeutic options (like methadone) together with the advancing availability and access to partial agonists such as buprenorphine.

## Methods

We conducted interrupted time-series analyses to assess the impacts of the removal of the federal methadone exemption requirements and related coincident provincial policies on the number of OAT prescribers. We conducted multiple analyses from all Canadian provinces, except Prince Edward Island and Newfoundland, collectively representing approximately 98% of the Canadian population at the time of the intervention implementation [16]. Relevant data from the three territories (Nunavut, Northwest Territories, and Yukon) were not available for this analysis. Informed consent was not required as all data were aggregated, de-identified, and collected from existing provincial and national administrative databases. This study is reported following the STROBE reporting guideline.

### Data sources

For British Columbia and Manitoba, we collected monthly claims and formulary data from the National Prescription and Drug Utilization Information System (NPDUIS) via the Canadian Institute for Health Information. The NPDUIS contains prescription claims-level data, and for British Columbia and Manitoba includes all community pharmacy claims (i.e. publicly and privately financed) and from all healthcare professionals (e.g. physicians and nurse practitioners). NPDUIS is widely used for evaluation, planning, and research purposes in Canada [17]. Relevant OAT prescribing data were not available for other provinces through NPDUIS.

We obtained monthly counts of prescribers in Ontario from the Narcotics Monitoring System housed at ICES (formerly the Institute for Clinical Evaluative Sciences), which captures all dispensing of controlled substances (including all buprenorphine and methadone prescribing), regardless of payer and prescriber profession in the province. ICES is an independent, non-profit research institute whose legal status under Ontario’s health information privacy law allows it to collect and analyze health care and demographic data, without consent, for health system evaluation and improvement. Datasets in Ontario were linked using unique encoded identifiers and analyzed at ICES [18].

Monthly data for all buprenorphine and methadone prescribers in Nova Scotia were obtained from Nova Scotia Health. Data pertaining to the utilization of monitored drugs are collected from community pharmacies and housed in the Nova Scotia Prescription Monitoring Program (NSPMP) database. These data are used at regional and provincial levels to inform prescribing practices, research, education initiatives and other stakeholder (e.g. regulatory colleges) activities [19].

For Alberta, Saskatchewan, Quebec, and New Brunswick, we collected monthly counts of physician-only prescribing of methadone and/or buprenorphine from IQVIA Canada Xponent (June 2016 – June 2022). IQVIA Canada Xponent data for Ontario and Nova Scotia were also collected to support secondary analyses. This database collects prescriber-level data from community pharmacy dispensations covering 57% of dispensations in Alberta and Ontario, 55% in Saskatchewan, 74% in Quebec, 66% in New Brunswick, and 63% in Nova Scotia, as of December 2021. IQVIA uses an internally validated proprietary geospatial projection algorithm to estimate all community pharmacy dispensations.

### Outcomes

Each data source provided monthly numbers of: (1) prescribers of methadone only, (2) prescribers of both methadone and buprenorphine, and (3) prescribers of buprenorphine only (for any kind of buprenorphine formulation used as OAT), each with a one year lookback period to determine prescribing status. The primary outcome was the monthly count of the overall number of prescribers (all three categories). Since the policy change might be expected to impact methadone prescribing only, as a secondary analysis we examined changes in the monthly number of methadone prescribers (prescribers of methadone only, plus prescribers of methadone and buprenorphine, not prescribers of buprenorphine only).

### Statistical analysis

We used descriptive statistics to capture the overall change in the rate of prescribers in each province. Given the large discrepancies in population size across provinces, we used contemporaneous provincial population estimates from Statistics Canada to determine the number of prescribers per 100,000 provincial residents. To determine the rate of change and the association of OAT policy changes with the outcomes, we used an interrupted time-series analysis that captured the changes in the numbers of methadone and/or buprenorphine prescribers between June 2017 and May 2019 — one year prior and one year post the policy intervention.

Interrupted time series analysis is an increasingly popular quasi-experimental method for comparing before and after an intervention or interruption. Interrupted time series can identify step changes (immediate effect), slope changes (sustained/gradual effect), and both, while accounting for potential confounders like seasonality and serial correlation. We included dummy variables for sharp peak or trough months in the regression models and correlated residuals were modeled as autoregressive of order 2 (AR(2)). The goodness of fit of each model was assessed by inspecting the fitted residuals for normality and white noise.

For our primary analysis, we used May 2018 as the interruption month. Since the policy was announced in late May, we conducted a sensitivity analysis using June 2018 as the interruption period. For Ontario and Nova Scotia we had access to two data sets, and elected to use the more comprehensive data sets in terms of percentage of claims and types of providers (ICES and NSPMP, respectively) for the primary analyses.

Hypotheses for the expected changes in the number of prescribers were based on the federal methadone exemption removal and any relevant coincident provincial regulatory changes (whether or not they were directly tied to the federal change). The provincial policy changes were determined by comprehensively reviewing and analyzing regulatory college documents, statements and any available reports pertaining to OAT, and were verified by local expert review. These provincial level changes are comprehensively documented elsewhere [20] and are summarized in Supplementary Appendix 1. Analyses were performed separately for each province. All tests were 2-sided with a statistical significance set at *p* < 0.05.

## Results

### Rate of change through the study period

Throughout the study period, all provinces had positive growth in the absolute number of OAT prescribers and in the number of prescribers normalized to the number of provincial residents (Table 1).

**Table 1:** Total number, number per 100,000 residents, and rate of change of all opioid agonist therapy and methadone prescribers through study period (June 2017 through May 2019), all provinces

Province	Number of all OAT prescribers June 2017 (per 100,000 residents)	Number of all OAT prescribers, May 2019 (per 100,000 residents)	Percent change (per 100,000 residents)	Number of methadone prescribers June 2017 (per 100,000 residents)	Number of methadone prescribers May 2019 (per 100,000 residents)	Percent change (per 100,000 residents)
BC	883 (18.0)	1315 (26.0)	48.9 (44.2)	621 (12.7)	869 (17.2)	39.9 (35.5)
AB	240 (5.7)	511 (11.8)	112.9 (107.2)	113 (2.7)	128 (2.9)	13.3 (10.2)
SK	27 (2.4)	50 (4.3)	85.2 (81.5)	7 (0.6)	19 (1.6)	171.4 (166.0)
MB	65 (4.9)	80 (5.9)	23.1 (19.8)	58 (4.4)	67 (4.9)	15.5 (12.4)
ON	1204 (6.9)	1792 (12.4)	48.8 (44.1)	461 (3.3)	537 (3.7)	16.5 (12.8)
QC	212 (2.6)	386 (4.6)	82.1 (77.9)	163 (2.0)	231 (2.7)	41.7 (38.5)
NB	42 (5.5)	65 (8.4)	54.8 (52.9)	36 (4.7)	46 (5.9)	27.8 (26.3)
NS	130 (13.7)	173 (17.9)	33.1 (30.6)	103 (10.9)	124 (12.8)	20.4 (18.1)

BC: British Columbia, AB: Alberta, SK: Saskatchewan, MB: Manitoba, ON: Ontario, QC: Quebec, NB: New Brunswick, NS: Nova Scotia

At the beginning of the study period, there were large differences across provinces in the number of OAT prescribers per 100,000 residents, with British Columbia and Nova Scotia having the highest rates at 18.0 and 13.7, respectively, while Quebec and Saskatchewan had the lowest rates at 2.4 and 2.6, respectively. In terms of the rate of growth, there was again a large range across provinces with Alberta having the largest percent growth of the OAT prescribing workforce through the study period at 107.2% and Manitoba having the least with a 19.8%, normalized to the number of residents.

There was similar positive growth in the number of methadone prescribers per 100,000 residents for all provinces. With the exception of Saskatchewan, however, the growth rate for methadone prescribers was lower for all provinces compared to the changes for all OAT prescribers. In the case of Alberta, the rate differential was greater than 10-fold in favor of all prescribers compared to methadone prescribers.

### Interrupted time series analyses

**Fig. 1:**
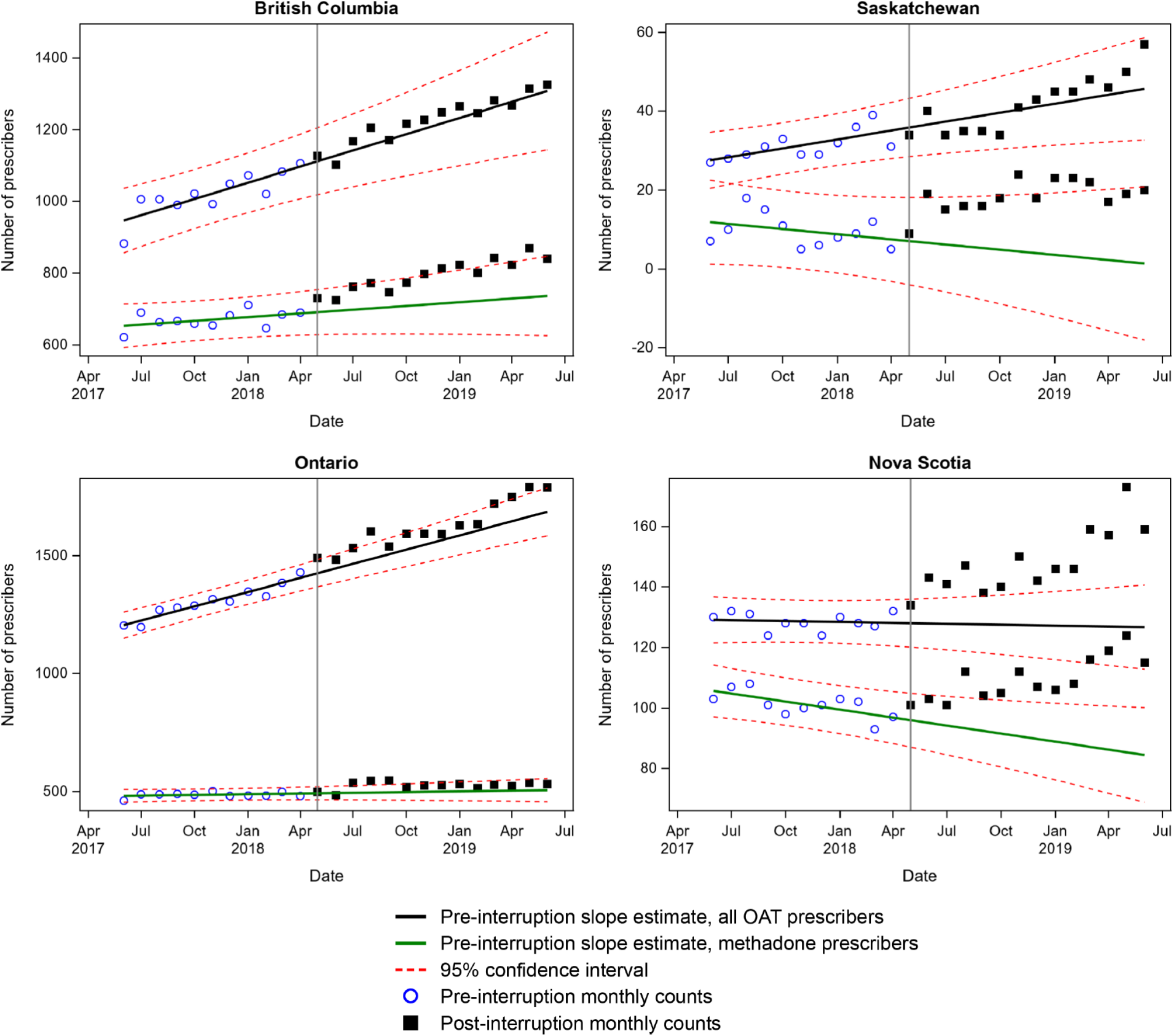
Interrupted time series for provinces with positive slope or step changes post May 2018 interruption (British Columbia, Saskatchewan, Ontario, Nova Scotia)

**Fig. 2:**
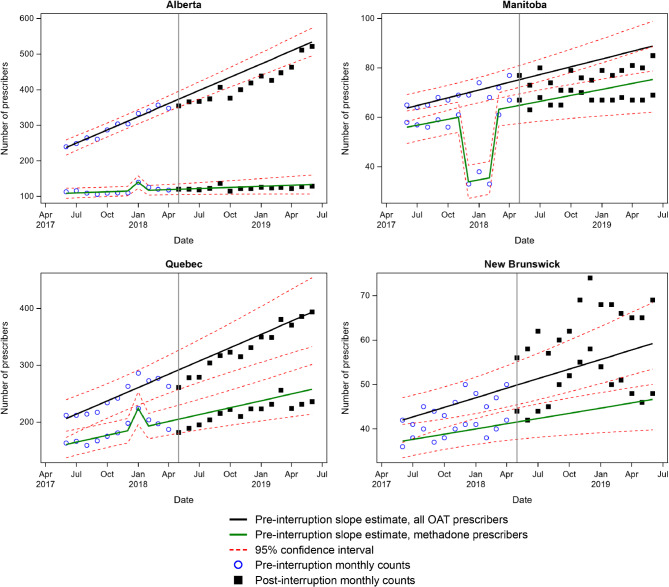
Interrupted time series for provinces with no or negative slope or step changes post May 2018 interruption (Alberta, Manitoba, Quebec, New Brunswick)

There were changes in either the primary or secondary outcome for each province coincident with the removal of the methadone exemption (Table 2; Figs. 1 and 2). However, there was variability across provinces in the nature and direction of this change. Positive changes reflect increases in the number of prescribers and negative changes reflect decreases. Sensitivity analyses using June 2018 as the interruption month are consistent with the trends reported below except where noted in the text (Supplementary Appendix 2).

**Table 2:** Interrupted time series results summarizing the association of the removal of the federal methadone exemption on number of monthly opioid agonist therapy prescribers, by province (main analysis with May 2018 as the interruption month)

Province		All OAT prescribers		Methadone prescribers	
	Model component	Parameter estimate (95% CI)	p value	Parameter estimate (95% CI)	p value
BC	Pre-interruption slope	12.6 (10.1, 15.1)	< 0.01	2.6 (0.8, 4.4)	0.01
	Post-interruption step change	31.4 (9.8, 53.0)	0.01	36.5 (20.8, 52.2)	< 0.01
	Post-interruption slope change	2.0 (-0.9, 4.9)	0.20	7.4 (5.2, 9.6)	< 0.01
AB	Pre-interruption slope	12.2 (9.3, 15.1)	< 0.01	1.0 (-0.0, 1.9)	0.06
	Post-interruption step change	-29.7 (-54.2, -5.2)	0.03	1.1 (-7.3, 9.5)	0.79
	Post-interruption slope change	-0.3 (-3.8, 3.2)	0.90	-0.6 (-1.8, 0.06)	0.38
SK	Pre-interruption slope	0.8 (0.5, 1.2)	< 0.01	-0.5 (-1.1, 0.1)	0.15
	Post-interruption step change	-5.2 (-9.5, -0.9)	0.02	7.3 (1.6, 13.0)	0.02
	Post-interruption slope change	0.7 (0.1, 1.3)	0.03	1.1 (0.03, 1.9)	0.01
MB	Pre-interruption slope	1.0 (0.6, 1.4)	< 0.01	0.8 (0.4, 1.2)	< 0.01
	Post-interruption step change	-0.4 (-3.1, 2.3)	0.73	2.5 (-0.4, 5.4)	0.12
	Post-interruption slope change	-0.5 (-0.9, -0.1)	0.01	-0.6 (-0.9, -0.2)	< 0.01
ON	Pre-interruption slope	20.3 (13.8, 26.8)	< 0.01	0.5 (-2.4, 3.4)	0.71
	Post-interruption step change	40.8 (-11.7, 93.3)	0.14	30.6 (6.7, 54.5)	0.02
	Post-interruption slope change	3.2 (-5.0, 11.4)	0.44	0.5 (-3.0, 4.0)	0.78
QC	Pre-interruption slope	7.6 (5.4, 9.8)	< 0.01	4.1 (1.9, 6.3)	< 0.01
	Post-interruption step change	-25.4 (-43.8, -7.0)	0.01	-14.6 (-32.2, 3.0)	0.12
	Post-interruption slope change	2.2 (-0.5, 4.9)	0.12	0.0 (-2.5, 2.5)	0.99
NB	Pre-interruption slope	0.8 (0.02, 1.6)	0.04	0.66 (-0.2, 1.6)	0.17
	Post-interruption step change	6.9 (1.2, 12.6)	0.03	2.9 (-2.2, 8.0)	0.28
	Post-interruption slope change	0.2 (-0.8, 1.2)	0.70	-0.2 (-1.6, 1.2)	0.75
NS	Pre-interruption slope	-0.2 (-1.2, 0.8)	0.73	-0.8 (-1.4, -0.2)	< 0.01
	Post-interruption step change	5.0 (-3.6, 13.6)	0.26	1.7 (-3.8, 7.2)	0.53
	Post-interruption slope change	2.3 (0.9, 3.7)	< 0.01	2.2 (1.4, 2.9)	< 0.01

BC: British Columbia, AB: Alberta, SK: Saskatchewan, MB: Manitoba, ON: Ontario, QC: Quebec, NB: New Brunswick, NS: Nova Scotia

### Positive slope or step changes

Saskatchewan and Nova Scotia both had significant upward slope changes post-May 2018 for both the primary (number of all OAT prescribers) and secondary (number of methadone prescribers) outcomes (Fig. 1). In both provinces, at the interruption point, there was a significant inflection from a negative slope (monthly loss in number of prescribers) to a positive slope (monthly gain in number of prescribers). Nova Scotia did not demonstrate any step changes, while Saskatchewan had a negative step change for the overall number of prescribers, but a positive step change for the number of methadone prescribers. Notably, in our secondary data set for Nova Scotia which included only physician prescribing, there were no significant changes in the overall number of OAT prescribers, and a significant negative slope change in the number of methadone prescribers (Supplementary Appendix 3).

Ontario showed no change in the primary outcome, but showed a significant upward step change with no slope change in the number of methadone prescribers (Fig. 1). British Columbia also had a significant upward step change in the number of methadone prescribers which was large enough to drive a significant upward step change in the overall number of prescribers. BC also had a significant positive slope change in the number of methadone prescribers post interruption.

### No slope or step changes

Quebec and New Brunswick both had significant step changes in the overall number of prescribers in May 2018 (Fig. 2). However, neither of these changes were significant in our sensitivity analysis using June 2018 as the interruption date (Supplementary Appendix 2). There were no other significant changes for these provinces.

### Negative slope or step changes

Manitoba was the only province to demonstrate a significant negative slope change, seen for both outcomes. There was no significant step change for this province (Fig. 2).

Alberta had a significant negative (significant) step and negative (non-significant) slope change in the number of all OAT prescribers. This province showed no other significant changes related to the interruption.

## Discussion

### Summary

In this first cross provincial comparison of the numbers of OAT prescribers using multiple provincial data sets, we have documented at least four important findings. First, there is a wide range in the number of OAT prescribers across provinces with a 7.5 fold difference between the highest and lowest provinces, normalized to the number of residents. This difference is even larger for methadone prescribers with the number per 100,000 residents in British Columbia being 10.8 times higher than that of Saskatchewan. Second, we identified an encouraging trend of significant increases in the number of OAT prescribers, including methadone prescribers, in each province over the study period. Third, there is substantial variability in the magnitude of this change with the fastest growing province growing at 5.4 fold the rate of the slowest growing province. Finally, we identified very distinct responses to the removal of the methadone exemption across the provinces ranging from clearly positive to possibly negative effects of this federal policy intervention.

### Interpretation

While the national discourse often refers to a *Canadian* drug toxicity crisis, important differences exist across the provinces and territories, particularly in the rates of drug-related harms and drivers of those harms such as the relative presence of fentanyl and its derivatives [14]. This is comparable to significant state-level variation seen in the US [21]. This study reinforces and advances our understanding that there are also important cross-jurisdictional differences in terms of systems of care for OUD and regimes governing OAT prescribing. These differences must be accounted for in federal policies responding to opioid-related harms and in subnational policy learning in order to maximize the policies’ intended beneficial effects and mitigate unintended negative consequences.

Understanding provincial differences with respect to OAT policies is essential for interpreting these data and much work needs to be done to elucidate these distinct systems of care and policy regimes. While some of our previous work in this area [20] has begun to do this and may help to explain some of the observed patterns, we did not identify any *consistent* patterns across provinces in terms factors such as geography (e.g. Western versus Eastern provinces), population (e.g. more versus less populous provinces), or pre-policy conditions (such as a saturation effect from a relatively high proportion of OAT prescribers). As such, we provide provisional speculations for the observed trends by similarly patterning pairs of provinces below. Quebec and New Brunswick already had relatively liberal regimes for OAT prescribing, with New Brunswick often drawing from Quebec’s provincial college standard. For example, prior to the removal of the federal exemption and unlike most other provinces across the country, Quebec had no mentorship nor continuing medical education requirements for methadone prescribing, and neither province had auditing or practice review requirements. Likewise, New Brunswick had allowed nurse practitioners to prescribe in September of 2014, a policy change which in the US has resulted in expansions of the OAT workforce. Importantly, both provinces also have amongst the lowest rates of drug toxicity harms in the country [14]. Given these antecedents, there may have been limited opportunity for the federal policy intervention to have a significant effect in these provinces.

Similarly, British Columbia in June 2017 had already shifted oversight of OAT prescribing away from a regulatory regime with the College of Physicians and Surgeons of BC to a more educational approach with the BC Centre for Substance Use, and had been the only province to have done so [20]. It was also the first province to declare overdose deaths a public emergency, which it did in 2016 [5, 22]. Likewise, coincident with the methadone exemption removal, in June 2018, BC made physician billing changes to make OUD care more remunerative [23]. This collection of changes may have primed the province to be able to take advantage of the federal removal of the methadone exemption and thus significantly increase its workforce for OAT delivery, including specifically methadone delivery. For Ontario, it was only in March 2021, well into the health system and drug toxicity changes wrought by the COVID-19 pandemic, that the provincial medical regulator’s methadone policy was rescinded [24], so the observed effect of a significant increase in methadone prescribers was likely not tied directly to explicit provincial regulatory policy and may represent a direct effect of the federal policy change.

For Nova Scotia, coincident with, and possibly related to the removal of the methadone exemption, was an expansion of prescribing to nurse practitioners [25]. Since the improvements in number of prescribers were noted only in the NSPMP data set (which includes all prescribers) and in fact an opposite effect was noted in the IQVIA Canada Xponent data set (MDs only), the driving force for the change in trajectory of number of prescribers may have been the allowance for NP prescribing. However, it should be noted that the number of NPs compared to the number of physicians in that province is quite low [26]. Saskatchewan merits further study as it demonstrates a similar pattern as Nova Scotia, however this province only expanded OAT prescribing to NPs in March 2019, after the interruption period, and these prescribers are not captured in the IQVIA Canada Xponent data set used for this analysis.

Alberta had by far the fastest growth trajectory amongst all provinces, which is relevant since it also has amongst the highest rates of drug toxicity harms [14]. Most of this increase was driven by increases in the numbers of buprenorphine only prescribers. There were minimal changes in medical prescribing regulations as a result of the federal exemption removal, and thus it is not surprising that there were no significant changes in the rate of the workforce expansion and a comparatively sluggish growth in the number of methadone prescribers. Alberta did introduce NP methadone prescribing in July 2018 [27]. Since our data set for this province only includes MD prescribing, the direct effects of this policy change are not well captured here, though the non-significant decrease in slope post May 2018 might reflect a shift of prescribing from MDs to NPs.

Finally, Manitoba, with high rates of drug toxicity harms compared to the Eastern provinces, has the most concerning pattern. The expansion rate of OAT prescribers was the lowest of all provinces, was significantly negatively impacted by the federal exemption removal, and the province had already enacted other strategies for workforce expansion with NP prescribing of methadone as far back as June 2002 [28]. Importantly, the province has made no substantive changes to its medical regulatory regime for methadone since the federal exemption removal and could stand to benefit from policy learning from provinces which have made important changes that have resulted in significant OAT workforce expansions. In 2023, with federal funding support, the provincial college did release a revised and comprehensive OAT practice manual [29], the impact of which is yet to be determined.

### Implications

In France, from the mid 1990s to the mid 2000s, there was a phenomenal expansion of access to OAT, with one report estimating less than 3% of people with OUD receiving buprenorphine or methadone in 1995 but then approximately 67% receiving OAT by 2006. This expansion is attributable to federal policies that removed regulatory barriers to prescribing buprenorphine particularly in primary care settings, and that made methadone more accessible through regulated specialized treatment centres [30]. While this “French Model” has continued to evolve over the subsequent decades [31], an important lesson from France is that federal policy changes can drive national level changes in access to care – especially when healthcare provision *and* drug regulation are both federal responsibilities [32].

This study clearly identifies that the Canadian experience has been different: distinct provincial health professional regulation and healthcare delivery regimes can have a kaleidoscoping effect on federal drug regulation policy change. This has clear implications for comparable jurisdictions such as the US with similar opportunities for federal policy change, such as the removal of the X waiver, but who also have substantial state-level variability in opioid treatment regulation [33]. Other federalist systems facing increasing rates of harms from opioids and contemplating various response strategies, such as Australia, will face similar complexities as Canada — suggesting important opportunities for cross-jurisdictional policy exchange and learning.

Our previous research in Canada has identified that OAT knowledge networks, and possibly policy networks, are relatively dominated by the more populous provinces of BC and Ontario. More than half of respondents to the national consultation on the federal methadone exemption policy were from just three provinces: BC, Alberta, and Ontario [34]. Likewise, the national discourse of the drug toxicity crisis has shifted to a focus on harms in Western provinces like BC [35]. In some ways this is appropriate because of the substantially higher rates of harms in these provinces and the greater absolute harms because of the larger populations. At the same time, this may shift federal and local attention away from provinces with smaller populations and lower rates of harms that nonetheless need nuanced and localized strategies to improve care for people living with OUD. Federal policy responding to issues of national policy relevance, such as drug toxicity harms, needs to be responsive, perhaps disproportionately so, to compensate for this relative lack of attention and resourcing available to less populous provinces. This analysis has identified specifically Manitoba as a less populous province with relatively high rates of harms and a negative response to the federal policy change.

Building on the current study, there are several further areas of research needed. Patient-level impacts, such as realized access to OAT, retention in treatment, and effects on toxicity harms, need further elucidation. This analysis demonstrated a 44.1% increase in the number of OAT prescribers in Ontario, while rates of opioid-related morbidity and mortality increased over the same time period from 10.6 per 100,000 in June 2017 to 13.7 per 100,000 in May 2019, or a 29% increase. Similar trends can be seen for BC [36]. However, in Ontario, about one third of opioid-related deaths are among people without evidence of OUD [37]. As such, provincial and national estimates of OUD prevalence are needed to determine the underlying need for OUD care services and appropriate responses. Furthermore, further provincial-level comparative policy analysis regarding OUD care and OAT provision is required to understand the specific policy trajectories in each province and how they interrelate. Specifically, key informant interviews with policy makers, health administrators, clinicians, and people who use drugs can provide a foundation for explaining the trends observed in this study. Likewise, the COVID-19 pandemic dramatically disrupted both the street drug supply and health system responses to the drug toxicity crisis, including provision of OAT. Further research is required to better characterize and compare how federal and provincial policy responses during and responsive to the pandemic have impacted the OAT prescriber workforce, realized OAT access, and appropriateness of OAT prescribing.

### Strengths and limitations

This study achieved a comprehensive review of OAT prescribing workforce trends from provinces covering the required time period and covering a large proportion of the Canadian population. To do this, we collected data from four distinct administrative data systems, at provincial and national levels, and from public and private sources. The number of different data sets and their distinct characteristics, such as variable coverage rates and variable inclusion of medical versus nursing prescribers, sets some limits on direct comparisons across provinces. For example, NPDUIS and IQVIA Canada Xponent data may underestimate the number of prescribers compared to the comprehensive provincial prescription drug monitoring programs of Ontario and Nova Scotia. This is reflective of the larger challenge of conducting national and comparative provincial analyses in Canada, noted acutely in response to the national crisis of drug toxicity deaths [38]. For the less populous provinces and provinces with relatively limited data sets, statistically significant changes should be interpreted with caution, as even small absolute changes may result in statistically significant changes on a background of low absolute numbers of prescribers. Likewise, comparisons across provinces may be interpreted with caution given the current dearth of estimates of OUD prevalence across provinces. Since data were not available, we cannot make any statements about the OAT prescribing workforces in the three Canadian territories.

Interrupted time series analysis is a rigorous method appropriate for making causal inferences especially with respect to policy changes. However, our ability to make causal inferences in this case is limited by several factors. In several provinces there were coincident provincial regulatory changes (e.g. expanding prescribing rights to NPs) that may or may not have been related to planning around expected federal-level policy initiatives such as the exemption removal. While our previous scholarship has begun to characterize regulatory and policy differences across provinces, more in-depth research on the policy histories and trajectories of each province is required to attribute workforce changes to specific policy interventions. Similarly, the selection of May 2018 as the interruption month is contestable given the time required for policy effects to wend their way through the health workforce. We mitigated this limitation by conducting a sensitivity analysis using June 2018 as the interruption period, and also by visually examining the time series for possible lagging step or slope changes.

## Conclusions

Federal drug regulation may be an attractive policy target to achieve widespread and rapid change, especially in response to large-scale health crises such as catastrophic drug toxicity harms and mortality occurring in countries like the US and Canada. However, especially in federalist health systems, antecedent subnational (state and provincial) regulations and policies have the potential to amplify, nullify, or even reverse the intended consequences of such federal policy maneuvers. There is a greater need to examine the mechanisms of these ripple effects and support policy learning and exchange across subnational jurisdictions.

### Electronic supplementary material

Below is the link to the electronic supplementary material.

Supplementary Material 1

Supplementary Material 2

Supplementary Material 3

## Data Availability

Data for this study were obtained by the authors with permission from Canadian Institute for Health Information, ICES, Nova Scotia Health, and IQVIA Canada.
