# Supplementary material for: Federal opioid agonist therapy policy: interrupted time series analysis of the impact of the methadone exemption removal across eight provinces in Canada
Source: BMC Health Serv Res. 2024 Aug 5;24:893. doi: 10.1186/s12913-024-11281-9 (PMC11302312; doi:10.1186/s12913-024-11281-9)
Supplement: Supplementary file 1 — Supplementary Material 1 [file 12913_2024_11281_MOESM1_ESM.docx]

Supplementary Appendix 1: Provincial OAT regulatory policies pre- and post- May 2018 removal of the methadone exemption and hypothesized change in number of methadone prescribers. BC: British Columbia, YT: Yukon Territory, AB: Alberta, SK: Saskatchewan, MB: Manitoba, ON: Ontario, QC: Quebec, NB: New Brunswick, NL: Newfoundland, NS: Nova Scotia, PE: Prince Edward Island.

| **Juris-diction** | **Initial education and training** | | **Mentorship or preceptorship** | | **Regular renewal or continuing education and training** | | **College registration** | | **Auditing or practice review** | | **Hypothesized direction of change in number of methadone prescribers post-exemption removal** |
| --- | --- | --- | --- | --- | --- | --- | --- | --- | --- | --- | --- |
|  | Pre | Post | Pre | Post | Pre | Post | Pre | Post | Pre | Post |  |
| BC^a^ | **•** | **•^*^** | **•** | **•^*^** | **•** |  | **•** | **•^*^** |  |  | No change |
| YT | **•** | **•** | **•** | **•** | **•** | **•** | **•** | **•** |  |  | No change |
| AB | **•** | **•** | **•** | **•** | **•** | **•** | **•** | **•** | **•** | **•** | No change |
| SK^b^ | **•** | **•** | **•** | **•** | **•** | **•** | **•** | **•** | **•** | **•** | No change |
| MB | **•** | **•** | **•** | **•** | **•** | **•** | **•** | **•** | **•** | **•** | No change |
| ON^c^ | **•** |  | **•** |  | **•** |  | **•** | **•** | **•** | **•** | No change |
| QC^d^ | **•** |  |  |  |  |  | **•** |  |  |  | Increase |
| NB | **•** |  | **•** |  | **•** |  | **•** |  |  |  | Increase |
| NL | **•** |  | **•** |  | **•** |  | **•** |  |  |  | Increase |
| NS | **•** |  | **•** |  | **•** |  | **•** |  | **•** |  | Increase |
| PE | **•** | **•** |  |  | **•** |  | **•** |  | **•** |  | Increase |

**•** Indicates required to prescribe in jurisdiction ^a^ Removed requirement for section 56 exemption in 2016 ^b^ Requires continuing medical education with no regular renewal ^c^ Removed education/training requirements in March 2021 ^d^ Required to name a mentor willing to support if necessary
*Applies only to those with no previous section 56 exemption prior to removal or no prescribing within the past 3 years
