# Supplementary material for: Federal opioid agonist therapy policy: interrupted time series analysis of the impact of the methadone exemption removal across eight provinces in Canada
Source: BMC Health Serv Res. 2024 Aug 5;24:893. doi: 10.1186/s12913-024-11281-9 (PMC11302312; doi:10.1186/s12913-024-11281-9)
Supplement: Supplementary file 2 — Supplementary Material 2 [file 12913_2024_11281_MOESM2_ESM.docx]

Supplementary Appendix 2: Interrupted time series results summarizing the association of the removal of the federal methadone exemption on number of monthly opioid agonist therapy prescribers, by province (sensitivity analysis - June 2018 interruption date). BC: British Columbia, AB: Alberta, SK: Saskatchewan, MB: Manitoba, ON: Ontario, QC: Quebec, NB: New Brunswick, NS: Nova Scotia.

| Province |  | All prescribers | | Methadone prescribers | |
| --- | --- | --- | --- | --- | --- |
|  | Model component | Parameter estimate  (95% CI) | p value | Parameter estimate  95% CI | p value |
| BC | Pre-interruption slope | 13.3 (11.2, 15.4) | <.01 | 3.9 (1.9, 5.9) | <.01 |
|  | Post-interruption step change | 33.5 (13.2, 53.8) | <.01 | 37.4 (18.1, 56.7) | <.01 |
|  | Post-interruption slope change | 0.8 (-1.9, 3.4) | 0.55 | 5.7 (3.2, 8.2) | <.01 |
| AB | Pre-interruption slope | 11.8 (8.1, 15.4) | <.01 | 1.1 (0.3, 1.9) | .01 |
|  | Post-interruption step change | -31.6 (-54.2, -9.0) | .01 | 0.91 (-7.0, 8.8) | .82 |
|  | Post-interruption slope change | 0.6 (-2.5, 3.7) | 0.72 | -0.72 (-1.7, 0.3) | .18 |
| SK | Pre-interruption slope | 0.80 (0.4, 1.2) | <.01 | -0.40 (-1.0, 0.2) | .19 |
|  | Post-interruption step change | -5.7 (-9.2, -2.2) | <.01 | 9.4 (4.0, 14.8) | <.01 |
|  | Post-interruption slope change | 0.86 (0.4, 1.3) | <.01 | 0.73 (0.0, 1.5) | <.07 |
| MB | Pre-interruption slope | 1.1 (0.9, 1.3) | <.01 | 0.9 (0.6, 1.2) | <.01 |
|  | Post-interruption step change | -2.5 (-4.9, 0.0) | .06 | 1.1 (-1.7, 3.9) | 0.46 |
|  | Post-interruption slope change | -0.5 (-0.8, -0.2) | <.01 | -0.7 (-1.0, -0.4) | <.01 |
| ON | Pre-interruption slope | 23.5 (16.9, 30.1) | <.01 | 0.8 (-1.2, 2.8) | .43 |
|  | Post-interruption step change | 6.5(-51.4, 64.4) | .83 | 32.9 (13.5, 52.3) | <.01 |
|  | Post-interruption slope change | 1.6 (-7.8, 11.0) | .75 | -0.3 (-2.9, 2.3) | .82 |
| QC | Pre-interruption slope | 6.0 (3.4, 8.6) | <.01 | 3.1 (0.9, 5.3) | .01 |
|  | Post-interruption step change | -5.2 (-28.0, 17.6) | .66 | -3.4 (-23.3, 16.5) | .74 |
|  | Post-interruption slope change | 3.4 (-0.4, 7.2) | .10 | 0.6 (-2.4, 3.6) | .71 |
| NB | Pre-interruption slope | 1.1(0.4, 1.8) | <.01 | 1.0 (0.0, 0.2) | .05 |
|  | Post-interruption step change | 4.1 (-2.3, 10.5) | .22 | -2.3 (-7.4, 2.8) | .38 |
|  | Post-interruption slope change | -0.1 (-1.1, 0.9) | .80 | -0.4 (-1.9, 1.1) | .64 |
| NS | Pre-interruption slope | 0.1 (-0.9, 1.1) | .87 | -0.7 (-1.2, -0.2) | .02 |
|  | Post-interruption step change | 5.4 (-3.6, 14.6) | .26 | 3.9 (-1.3, 9.1) | .16 |
|  | Post-interruption slope change | 2.0 (-1.3, 1.7) | .01 | 2.0 (1.3, 2.7) | <.01 |
