# Supplementary material for: Federal opioid agonist therapy policy: interrupted time series analysis of the impact of the methadone exemption removal across eight provinces in Canada
Source: BMC Health Serv Res. 2024 Aug 5;24:893. doi: 10.1186/s12913-024-11281-9 (PMC11302312; doi:10.1186/s12913-024-11281-9)
Supplement: Supplementary file 3 — Supplementary Material 3 [file 12913_2024_11281_MOESM3_ESM.docx]

Supplementary Appendix 3: Interrupted time series results summarizing the association of the removal of the federal methadone exemption on number of monthly opioid agonist therapy prescribers, by province (secondary analysis using secondary data sets). ON: Ontario, NS: Nova Scotia.

| Province |  | All prescribers | | Methadone prescribers | |
| --- | --- | --- | --- | --- | --- |
|  | Model component | Parameter estimate  95% CI | p value | Parameter estimate  95% CI | p value |
| ON (IQVIA Canada Xponent) | Pre-interruption slope | 8.7 (1.7, 15.8) | .02 | 0.2 (-1.7, 2.0) | .85 |
|  | Post-interruption step change | 10.3 (-37.6, 58.2) | .68 | 10.6 (-4.6, 25.9) | .19 |
|  | Post-interruption slope change | 9.2 (-0.7, 19.2) | .08 | 0.9 (-1.3, 3.1) | .44 |
| NS (IQVIA Canada Xponent) | Pre-interruption slope | 0.9 (-0.3, 2.1) | .14 | 1.1 (0.5, 1.7) | <.01 |
|  | Post-interruption step change | 2.1 (-7.0, 11.3) | .65 | 0.6 (-4.1, 5.2) | .81 |
|  | Post-interruption slope change | 0.1 (-1.5, 1.6) | .94 | -0.9 (-1.6, -0.1) | .03 |
